# Fulminant Myocarditis with SARS-CoV-2 Infection: A Narrative Review from the Case Studies

**DOI:** 10.1155/2024/9000598

**Published:** 2024-03-04

**Authors:** Ryohei Ono, Togo Iwahana, Kaoruko Aoki, Hirotoshi Kato, Sho Okada, Yoshio Kobayashi

**Affiliations:** Department of Cardiovascular Medicine, Chiba University Graduate School of Medicine, 1-8-1 Inohana, Chuo-ku, Chiba 260-8670, Japan

## Abstract

One of the severe complications of the severe acute respiratory syndrome coronavirus 2 (SARS-CoV-2) infection is myocarditis. However, the characteristics of fulminant myocarditis with SARS-CoV-2 infection are still unclear. We systematically reviewed the previously reported cases of fulminant myocarditis associated with SARS-CoV-2 infection from January 2020 to December 2022, identifying 108 cases. Of those, 67 were male and 41 female. The average age was 34.8 years; 30 patients (27.8%) were ≤20 years old, whereas 10 (9.3%) were ≥60. Major comorbidities included hypertension, obesity, diabetes mellitus, asthma, heart disease, gynecologic disease, hyperlipidemia, and connective tissue disorders. Regarding left ventricular ejection fraction (LVEF) at admission, 93% of the patients with fulminant myocarditis were classified as having heart failure with reduced ejection fraction (LVEF ≤ 40%). Most of the cases were administered catecholamines (97.8%), and mechanical circulatory support (MCS) was required in 67 cases (62.0%). The type of MCS was extracorporeal membrane oxygenation (*n* = 56, 83.6%), percutaneous ventricular assist device (Impella®) (*n* = 19, 28.4%), intra-aortic balloon pumping (*n* = 12, 12.9%), or right ventricular assist device (*n* = 2, 3.0%); combination of these devices occurred in 20 cases (29.9%). The average duration of MCS was 7.7 ± 3.8 days. Of the 76 surviving patients whose cardiac function was available for follow-up, 65 (85.5%) recovered normally. The overall mortality rate was 22.4%, and the recovery rate was 77.6% (alive: 83 patients, dead: 24 patients; outcome not described: 1 patient).

## 1. Background

The severe acute respiratory syndrome coronavirus 2 (SARS-CoV-2) infection or coronavirus disease 2019 (COVID-19) pandemic has been a global public health issue leading to significant morbidity and mortality worldwide [1, 2]. SARS-CoV-2 infection predominately results in an acute respiratory illness; however, sometimes cardiovascular complications arise, such as heart failure, pericardial effusion, and, rarely, myocarditis [3]. SARS-CoV-2 infection-related myocarditis has been reported since the beginning of the viral outbreak; fulminant myocarditis is a rare, yet life-threatening, variant with significant mortality, and often demands the emergent initiation of mechanical circulatory support (MCS) [3, 4]. Additionally, balancing infection protection and its treatment is challenging. Fulminant myocarditis due to SARS-CoV-2 infection is very rare and its characteristics still unclear. In this systematic literature review, we aimed to describe all cases of myocarditis associated with SARS-CoV-2 infection reported globally.

## 2. Methods

### 2.1. Study Design

We systematically reviewed the literature for reports of fulminant myocarditis associated with SARS-CoV-2 infection. This literature review was conducted in concordance with the guidelines provided by the Preferred Reporting Items for Systematic Reviews and Meta-Analysis (PRISMA) statement [5]. Registration of a review protocol was deemed unnecessary, as we used data presented in published literature for this study.

### 2.2. Eligibility and Exclusion Criteria

The included publications were full-length manuscripts retrieved with our search that contained data on one or more patients who acutely presented with myocarditis and recent SARS-CoV-2 infection, which was definitively diagnosed by any tests. Myocarditis was diagnosed by one or more of the following characteristics: clinically suspected myocarditis [6], elevated troponin levels and abnormal electrocardiograms, and impaired cardiac function on echocardiography and findings consistent with myocarditis on cardiac magnetic resonance (CMR) imaging (including myocardial edema or late gadolinium enhancement) or on endomyocardial biopsy (EMB) [7]. Moreover, fulminant myocarditis was defined as myocarditis with the new onset of heart failure with cardiogenic shock requiring ionotropic drugs or MCS, or histologically proven myocarditis with sudden death for which autopsy was available. Publications were first screened and excluded if they were written in languages other than English without an English interpretation. After the first screening, publications were excluded if any of the following conditions were met: not a case report or a human report; not a case of SARS-CoV-2 infection-related myocarditis; not a case of related myocarditis (for example, cases of acute coronary syndrome or pericarditis); not a case of fulminant myocarditis, using the definition provided above.

### 2.3. Search Strategy

We searched PubMed for all articles on myocarditis with SARS-CoV-2 infection published from January 1, 2020, to December 31, 2022, using the following keywords: (((((2019 novel coronavirus) OR (COVID-19)) OR (SARS-CoV-2)) OR (2019 ncov infection)) OR (2019 novel coronavirus)) AND (((cardiogenic shock) AND (myocarditis)) OR (fulminant myocarditis) OR (((extracorporeal membrane oxygenation) OR (Intra-aortic balloon pumping) OR (Impella)) AND (myocarditis))).

**Figure 1:**
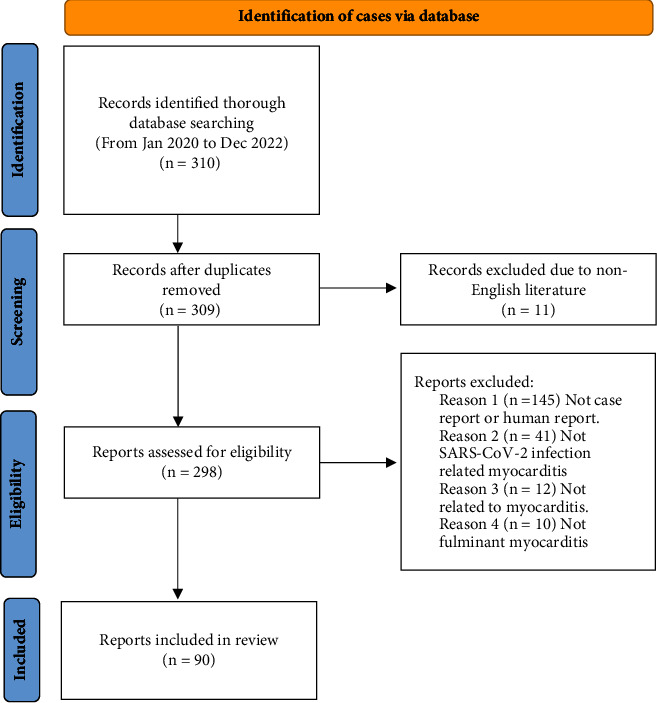
The PRISMA flowchart.

All articles retrieved from the systematic search were exported to EndNote Reference Manager (Version X9; Clarivate Analytics, Philadelphia, Pennsylvania, USA). All the identified publications were further screened for the inclusion and exclusion criteria by reading the full-text publications. The articles were assessed by two assessors (RO and TI) independently; if the two assessors' decision differed, a third assessor (HK) provided the final decision for inclusion. The PRISMA flowchart summarizes the results of our literature search (Figure 1).

### 2.4. Data Extraction Process

The included publications were analyzed for the authors' names, publication year, and patient-related data, namely, demographics, comorbidities, history of vaccination, clinical presentation, findings on echocardiography, arrhythmia, CMR data, biopsy findings, treatments, and outcomes.

## 3. Results

We identified a total of 108 patients from 90 studies relevant to fulminant myocarditis with SARS-CoV-2 infection (Tables 1 and 2) [8–97] of which 67 were male (62%) and 41 female (38%). The mean age of the patients was 34.8 ± 18.1 (range 0–72) years; thirty patients (27.8%) were ≤20-years old, whereas 10 (9.3%) were ≥60. Almost half the patients (*n* = 48) were previously healthy, and within the ones that presented major comorbidities, those included hypertension (*n* = 12), obesity (*n* = 11), diabetes mellitus (*n* = 8), asthma (*n* = 4), heart disease (*n* = 4), gynecologic disease (*n* = 4), hyperlipidemia (*n* = 3), and connective tissue disorders (*n* = 3); patient's characteristics were not described in detail in 21 cases. Only 4 patients received previous vaccination; among the 19 cases with available vaccination history, 2 patients received the first dose, 1 received two doses, and 1 received three doses. However, the vaccination history was not documented in most cases, as the vaccine itself was initially unavailable in several countries. No patients had received more than three doses of the vaccine. Excluding the 10 patients whose symptoms were not reported, fever (*n* = 51, 52.0%) was the most common symptom at initial presentation, followed by dyspnea or shortness of breath (*n* = 45, 45.9%), diarrhea (*n* = 20, 20.4%), chest pain (*n* = 20, 20.4%), cough (*n* = 19, 19.4%), vomiting (*n* = 17, 17.3%), and abdominal pain (*n* = 13, 13.3%). Vague symptoms such as asthenia (*n* = 9, 9.2%), fatigue (*n* = 9, 9.2%), weakness (*n* = 5, 5.1%), lethargy (*n* = 5, 5.1%), and loss of appetite (*n* = 3, 3.1%) were unusual. The median time from symptom onset to myocarditis diagnosis was 6 days (Interquartile range 3–9 days).

Myocarditis with concurrent pneumonia occurred in 43 cases (45%), of which 20 were in 2020 and 2021, and only 3 after 2021.

Among the 92 patients whose left ventricular ejection fraction (LVEF) on echocardiography at admission was available, 48 (52.2%) were classified as having LVEF ≤ 20%, 31 with 20 < LVEF ≤ 30% (33.7%), 7 with 30 < LVEF ≤ 40% (7.6%), 3 with 40 < LVEF ≤ 50% (3.3%), and 3 with 50% < LVEF (3.3%), which includes preserved or normal ejection fraction. The patients with 50% < LVEF were associated with the presence of ectopic wandering atrial pacemaker or asystole. Pericardial effusions were observed in 45 patients (65.2%) and left ventricular wall thickening was identified in 24 (40.7%).

Regarding arrhythmia, lethal arrhythmias, namely, ventricular tachycardia and ventricular fibrillation, occurred in 11 and 5 patients, respectively. Cardiac arrest, presented as pulseless electrical activity or asystole, occurred in 6 and 6 cases, respectively. Identified cardiac conduction defects included right bundle branch block (*n* = 5) and complete atrioventricular block (*n* = 4).

The diagnosis of myocarditis was made solely by CMR (*n* = 14, 13.0%), biopsy (*n* = 23, 21.3%), or both (*n* = 12, 11.1%), whereas the remaining cases (*n* = 659, 54.6%) were clinically diagnosed.

Antiviral treatment was administered in 35 cases, whereas immunomodulatory therapy was performed in 78; the most common immunomodulatory therapy was steroid administration (*n* = 72), followed by intravenous immunoglobulin (IVIG) (*n* = 38), tocilizumab (*n* = 13), and anakinra (*n* = 6).

Among the 93 patients whose catecholamine use history was available, most (*n* = 91, 97.8%) underwent catecholamine use. MCS was employed in 67 cases (62.0%). The type of MCS used was extracorporeal membrane oxygenation (ECMO) (*n* = 56, 83.6%), percutaneous ventricular assist device (Impella®) (*n* = 19, 28.4%), intra-aortic balloon pumping (IABP) (*n* = 12, 12.9%), or right ventricular assist device (RVAD) (*n* = 2, 3.0%); combination of devices occurred in 20 cases (29.9%). The average duration of MCS was 7.7 ± 3.8 days. Cardiac function recovered to normal (LVEF ≥ 50%) in 67 cases. Of the 76 surviving patients whose cardiac function was available for follow-up, 65 (85.5%) recovered normally.

Finally, the overall mortality rate was 22.4%, and the recovery rate was 77.6% (alive: 83 patients, dead: 24 patients; outcome not described: 1 patient). One patient underwent a heart transplant.

## 4. Discussion

In this systematic review, we summarized the features of fulminant myocarditis with SARS-CoV-2 infection, including patients' demographics, comorbidities, history of vaccination, symptoms, clinical characteristics, treatments, and outcomes. To our knowledge, this is the first comprehensive review analyzing all cases of fulminant myocarditis related to SARS-CoV-2 infection.

### 4.1. Patients' Clinical Characteristics

The incidence of acute myocarditis in the general population is estimated to be approximately 10–22 per 100,000 people [98, 99]. The estimation of the mean prevalence of SARS-CoV-2 infection-related acute myocarditis was reportedly between 0.0012 and 0.0057 among hospitalized patients with SARS-CoV-2 infection [3]. Although the incidence of fulminant myocarditis is less well-defined, the condition is considered quite rare. Our systematic review revealed that only 108 cases of fulminant myocarditis with SARS-CoV-2 infection were reported between 2020 and 2022.

The mean age of the 108 patients with myocarditis with SARS-CoV-2 infection was 35 years, and 62% of them were male. Myocarditis has been reported to occur more frequently in males, with a male to female ratio around 1.5 : 1–1.7 : 1; therefore, the current review was consistent with previous reports [100, 101]. Surprisingly, the case of a 3-day-old newborn with myocarditis was reported; if mothers do not possess antibodies against COVID-19, newborns can be infected with the virus [38]. Conversely, the incidence was not so high among the elderly. Myocarditis typically occurs between 3 and 9 days after the onset of COVID-19 symptoms. The time course of the occurrence of myocarditis was similar to other viral infections, such as influenza [102].

### 4.2. Pathophysiology and Comorbidities

The possible pathophysiology of COVID-19 myocarditis is thought to involve the direct invasion of cardiac myocytes by the SARS-CoV-2 virus, and indirect cardiac injury due to increased release of cytokines and inflammatory pathways [103, 104]. The densities of CD68+ macrophages and CD3+ lymphocytes have been reported to be relatively high in myocarditis, from the results of EMB; additionally, myocardial macrophage and lymphocyte densities displayed a positive correlation with the symptom duration of myocarditis [105]. Thus, cytokines and inflammatory pathways are likely to play key roles in myocarditis' pathogenesis.

Previous reviews described that patients with cardiovascular comorbidities, such as hypertension, diabetes, obesity, hyperlipidemia, and ischemic heart disease were at a higher risk of developing COVID-19 myocarditis [104]. The results of our analysis revealed that hypertension, obesity, and diabetes mellitus were the most common comorbidities among patients with SARS-CoV-2 infection-related “fulminant” myocarditis. The association between hypertension and inflammation is well-known; inflammatory responses increase the disease's severity and patients' complications [106]. Obesity is associated with adipose tissues, chronic low-grade inflammation, and immune dysregulation with hypertrophy and hyperplasia of adipocytes and overexpression of proinflammatory cytokines. Increased epicardial and pericardial thickness can be observed on echocardiography of patients with myocarditis and has been attributed to an increased amount of epicardial adipose tissue (EAT), a highly inflammatory reservoir with dense macrophage infiltration and increased levels of proinflammatory cytokines, such as interleukin 6 (IL-6) [107]. EAT could fuel COVID-19-induced cardiac injury and myocarditis [108]. The EAT volume, as well as the volume of visceral adipose tissue, is increased in obese patients; therefore, obesity is also one of the major risk factors for myocarditis [109].

### 4.3. Vaccination and Variant of the Virus

Regarding vaccination, myocarditis following vaccination has been reported, with an incidence of myocarditis/pericarditis of 4.5 per 100,000 vaccinations across all doses [110]. Our review revealed that most cases of fulminant myocarditis caused by COVID-19 did not receive vaccination; however, vaccination's number was limited, with the accumulation of more findings being expected in the future.

The incidence of concurrent myocarditis and pneumonia has decreased over time, probably because of the change of viral variant and the widespread use of vaccines. The severity of COVID-19 is milder with the Omicron variant, compared with Alpha and Delta variants, identified by whole genome sequencing. In addition, Omicron has difficulty replicating in the lungs compared to the Delta variant, which may explain the reduced respiratory impairment with the Omicron [111, 112].

### 4.4. Clinical Presentation

The most reported symptoms were fever, dyspnea, shortness of breath, chest pain, and cough. These are typical manifestations in myocarditis as well as in COVID infections; accordingly, reaching an appropriate diagnosis can be challenging [113].

Regarding LVEF at admission, more than 90% of the patients with fulminant myocarditis were classified as having heart failure with reduced ejection fraction (LVEF ≤ 40%). Notably, regardless of the severity of the acute myocardial injury, the cardiac function of most patients returned to normal if they survived; our review showed that 85.5% of the patients recovered to a normal cardiac function. In previous reports of acute cardiac injury in patients with SARS-CoV-2 infection, 89% of the patients presented a LVEF of approximately 67%, while 26% developed myocarditis-like scars [114]. The long-term effect of such cardiac injury data is still unknown, and waiting for the follow-up data is warranted.

The overall incidence of arrhythmia in patients with COVID-19 was reported as 16.8%, of which approximately 8.2% constituted atrial arrhythmias (atrial fibrillation or atrial flutter), 10.8% conduction disorders, 8.6% ventricular tachycardia (ventricular tachycardia, tachycardia/ventricular flutter/ventricular fibrillation), and 12% unclassified arrhythmias [115]. Our review revealed that lethal arrhythmias or cardiac arrest occurred in a total of 26 cases with fulminant myocarditis (24.1%), a rate higher than previously reported. These arrhythmias often required MCS, and 62% of the patients in our review received MCS.

### 4.5. Diagnosis

CMR and EMB are essential myocarditis diagnostic tests. However, due to the risk of infection, such were sometimes not performed in patients with COVID-19. Additionally, CMR is usually performed after myocarditis stabilization, in a subacute phase. According to the revised Lake Louise Criteria of 2018, CMR-based diagnosis of myocarditis is based on at least one T1-based criterion (increased myocardial T1 relaxation times, extracellular volume fraction, or late gadolinium enhancement) with the presence of at least one T2-based criterion (increased myocardial T2 relaxation times, visible myocardial edema, or increased T2 signal intensity ratio). Additionally, supportive criteria include the presence of pericardial effusion in cine CMR images or high signal intensity of the pericardium in late gadolinium enhancement images, T1-mapping or T2-mapping, and systolic left ventricular wall motion abnormality in cine CMR images [7]. Diagnosis of myocarditis using the Lake Louise Criteria has a 91% specificity and 67% sensitivity. CMR can be used as a primary diagnostic technique for screening COVID-19-associated myocarditis in the absence of contraindications [116].

EMB remains the gold standard invasive technique in diagnosing myocarditis, and, especially for fulminant myocarditis with a fatal outcome, autopsy is also an useful diagnostic tool [117]. The sequence of myocardial damage after SARS-CoV-2 infection obtained from autopsy reviews varied. Raman et al. reported that only four patients (5%) presented suspected cardiac injury in an early autopsy series of 80 consecutive SARS-CoV-2 positive cases; two patients had comorbidities and died of sudden cardiac death, one presented acute myocardial infarction, and another showed right ventricular lymphocytic infiltrates. These results suggested that extensive myocardial injury as a major cause of death may be infrequent [118]. Basso et al. investigated cardiac tissue from the autopsies of 21 consecutive patients with COVID-19 assessed by cardiovascular pathologists. Myocarditis (characterized as lymphocytic infiltration as well as myocyte necrosis) was seen in 14% of the cases, infiltration of interstitial macrophage in 86%, and pericarditis as well as right-sided ventricular damage in 19% [119]. Halushka and Vander Heide reviewed 22 publications that described the autopsy outcomes of 277 affected individuals. Lymphocytic myocarditis was mentioned in 7.2% of cases, however, only 1.4% met the strict histopathological criteria for myocarditis, implying that proper myocarditis was uncommon; such cases comprised autopsies from patients with COVID-19 without a definitive myocarditis diagnosis before death [120]. Our review showed that diffuse lymphocytic inflammatory infiltrates with edema was the most common finding, and a few cases were associated with eosinophilic infiltrations in patients with confirmed myocarditis with SARS-CoV-2 infection.

In addition, it is difficult for clinicians to differentiate myocarditis with pneumonia from myocarditis with acute pulmonary edema. The distinction between myocarditis with COVID-19 pneumonia and myocarditis with acute pulmonary edema is primarily based on imaging findings and laboratory markers. Both conditions often present with similar symptoms such as fever, cough, and dyspnea. However, patients with myocarditis and pneumonia often have imaging studies that show localized pulmonary infiltrates or consolidation. The hallmark of COVID-19 pneumonia is the presence of ground-glass opacities, typically with a peripheral and subpleural distribution. In addition, the involvement of multiple lobes, particularly the lower lobes, has been reported in most cases of COVID-19 pneumonia [121]. In contrast, myocarditis with acute pulmonary edema typically presents with bilateral alveolar infiltrates indicating fluid overload [121]. Elevated biomarkers of heart failure such as brain natriuretic peptide (BNP) or N-terminal pro-BNP also suggest myocarditis with pulmonary edema [113]. Ultimately, the distinction is made by a combination of symptoms, specific imaging features, and the presence of biomarkers to guide the appropriate management of each condition.

### 4.6. Treatment

The management of myocarditis with SARS-CoV-2 is currently controversial and not yet established. Both American and European guidelines propose a management similar to that of other viral myocarditis and heart failure treatment [122, 123]. Hospitalization is recommended for patients with confirmed myocarditis that is either mild or moderate in severity, ideally at an advanced heart failure center. Patients with fulminant myocarditis should be managed at centers with an expertise in advanced heart failure, MCS, and other advanced therapies [122]. European consensus suggested that escalation to MCS should be carefully weighed against the development of coagulopathy associated with COVID-19 and the need for specific treatments for acute lung injury, such as prone position; when MCS is required, ECMO should be the preferred temporary technique, because of its oxygenation capabilities [123].

Regarding the specific treatment of COVID-19-associated myocarditis, no compelling evidence exists to support the use of immunomodulatory therapy, including corticosteroids and IVIG [123]. However, some authors indicate a possible benefit of high-dose steroids and IVIG, as the condition can be considered an immune-mediated myocarditis. Corticosteroids are indicated when respiratory involvement is present and have been administered to patients who showed favorable clinical outcomes [124, 125]. For those with pericardial involvement, nonsteroidal anti-inflammatory drugs may be used to help alleviate chest pain and inflammation. Regarding IVIG in myocarditis not associated with COVID-19, a meta-analysis reported improved survival and ventricular function with its administration with corticosteroids, especially in acute fulminant myocarditis [126]. Other immunomodulatory therapies, such as tocilizumab and anakinra, are currently being studied for SARS-CoV-2-associated myocarditis [122, 127]. Regarding antiviral treatment, none demonstrated efficacy at reducing COVID-19 mortality [128]. In this review, remdesivir was employed in 14 cases, and four of them culminated in death. Lopinavir/ritonavir were used in 4 cases, all of which survived. As for MCS, a large retrospective review that analyzed 147 patients with a diagnosis of acute myocarditis treated with ECMO from 1995 to 2011 showed that survival to hospital discharge was 61%, confirming ECMO as a useful therapy in adults with myocarditis with cardiogenic shock and highlighting its high in-hospital mortality [129]. Inadequate aortic valve opening or lack of left ventricular support could occasionally occur with single ECMO therapy; therefore, those cases may require dual cardiac assist devices to ensure adequate ventricular unloading, such as ECMO with Impella® or with IABP.

### 4.7. Prognosis and Outcomes

Rathore et al. reported that approximately 38% of the patients with SARS-CoV-2 infection-related myocarditis required vasopressor support; out of 28 patients, 82% survived, whereas 18% died [117]. Furthermore, Urban et al. reported that death was the outcome in 11 out of 63 cases (17%) [130]. Our review showed that the overall mortality rate was 22.4%, and the recovery rate was 77.6%, which were worse outcomes than the previously reported, because of our focus on fulminant myocarditis. However, reported cases are usually severe and complicated, which may constitute a bias for reporting a higher mortality.

### 4.8. Limitations

This systematic review had several limitations. First, our study is retrospective and descriptive in nature. In some cases, the myocarditis diagnosis was based on clinical expertise. CRM image acquisition was not standardized and relied on local protocols. A possibility of publication bias also exists, in which fatal forms of SARS-CoV-2 infection-associated myocarditis may not have been reported or identified due to its challenging diagnosis. Additionally, only published data including inpatient cases were included in the study. Clinical evaluations such as subjective symptoms reporting and many of the objective values may vary. Lastly, the clinical workup was heterogeneous.

## 5. Conclusions

In conclusion, we reviewed previously reported cases of fulminant myocarditis with SARS-CoV-2 infection. We summarized an international experience with this severe condition that was accumulated for the last three years, since the start of this pandemic. We demonstrated that SARS-CoV-2 infection-associated fulminant myocarditis required MCS in 62% of the cases and resulted in death of one out of five patients, therefore demonstrating its high mortality. Conversely, most of the surviving patients recovered to normal systolic functions. Therefore, rapid bridging therapy including immunomodulatory therapies and/or MCS, if appropriate, may play an important role for improving outcomes in patients with fulminant myocarditis with SARS-CoV-2 infection.

## Figures and Tables

**Table 1:** Literature review of cases with fulminant myocarditis with SARS-CoV-2 infection.

Case	Author	Year (reference)	Age (years)	Sex	Comorbidities	Dose of vaccination	Initial symptoms	Time (symptoms to diagnosis of myocarditis)	Pneumonia	LVEF (%)	PE	LVWT	Arrhythmia	CMR	Biopsy findings	Catecholamine	Antiviral treatment	Immunomodulatory therapy	MCS	Duration of MCS use (days)	Cardiac recovery	Outcome
1	Noone et al.	2022 [8]	38	F	None	0	Cold-like symptoms and relapsing syncope	5	No	<20	Yes	Yes	None	—	—	Yes	—	—	VA-ECMO Impella CP	8	Fully	Alive
2	Hoang et al.	2022 [9]	42	F	ND	3	Chest pain, dyspnea, lethargy, and fever	8	No	25	No	No	VF	—	—	No	No	No	VA-ECMO	7	Fully	Alive
3	De Smet et al.	2022 [10]	25	M	None	2	Fever, abdominal pain, vomiting, and diarrhea	6	No	Moderately decreased LVEF	Yes	No	None	Moderately dilated left ventricle with moderately reduced systolic function, increased myocardial extracellular volume by T1-mapping, no focal myocardial LGE	—	Yes	No	Corticosteroid IVIG	No	—	Fully	Alive
4	Usui et al.	2022 [11]	44	F	None	0	Chest pain	5	No	10	Yes	Yes	None	Diffuse oedematous wall thickening with high signal intensity in T2-weighted images, LGE in the basal to the apical inferolateral mid-myocardial wall	—	Yes	Remdesivir	Corticosteroid baricitinib	VA-ECMO Impella CP	10	Fully	Alive
5	Ardiana and Aditya	2022 [12]	40	M	None	ND	Chest pain	3	No	ND	Yes	Yes	CAVB	—	—	Yes	No	—	IABP	6	Fully	Alive
6	Ya'Qoub et al.	2022 [13]	30	M	ND	ND	Heart failure symptoms	ND	ND	10	ND	ND	VF	—	—	ND	—	—	VA-ECMO	—	ND	ND
7	Ajello et al.	2022 [14]	49	M	None	ND	Fever and dyspnea	4	No	10	No	ND	ND	Diffuse increase of native T2 and native T1, no LGE	Lymphomonocytic inflammatory infiltrates with cardiomyocytes necrosis	Yes	No	No	Impella CP/5.0/RP	10	Fully	Alive
8	Asakura et al.	2022 [15]	49	M	None	ND	Chest pain	6	No	<20	No	Yes	None	Mild LGE on the epicardial side of the inferior wall of the heart base, mild high signal on T2-weighted MRI of the same area, mild high signal on T1-weighted MRI, mild fibrosis, and edema-like changes	Mild myocyte hypertrophy, some subendocardial fibrosis, and scattered cluster of differentiation 3 (CD3)-positive T cells	Yes	No	Methylprednisolone	Impella 5.0	10	Fully	Alive
9	Nakatani et al.	2022 [16]	49	M	None	1	Sore throat, chill, and fever	4	Yes	<20	ND	Yes	None	—	Mild lymphocytic infiltration and moderate to severe perivascular fibrosis with wall thickening of intramural arterioles	Yes	No	Methylprednisolone, IVIG	ECMO	5	—	Dead
10	Callegari et al.	2022 [17]	15	M	None	ND	Reduced appetite, gastroenteritis, mild dyspnoea, and dizziness	13	No	20	Yes	No	VT	No myocardial oedema or myocardial contrast enhancement	The histological results were not consistent with an acute/chronic lymphocytic, eosinophilic, or giant-cell myocarditis, or dilated cardiomyopathy	Yes	No	No	No	—	Fully	Alive
11	Phan et al.	2022 [18]	9	M	None	ND	Fever, cough, and sore throat	4	No	18	ND	ND	None	—	—	Yes	No	Dexamethasone	VA-ECMO	10	Fully	Alive
12	Carrasco-Molina et al.	2022 [19]	36	M	ND	ND	Dyspnea and chest pain	7	No	30	No	No	None	Myocardial inflammation	Lymphocytic inflammatory infiltrate (35 CD3+/mm^2^ lymphocytes), without myocyte necrosis or fibrosis	Yes	No	Methylprednisolone	No	—	Fully	Alive
13	Kohli et al.	2022 [20]	15	F	None	ND	Headache, vomiting, and fatigue	1	No	20 (LVSF)	No	No	AF	—	—	Yes	—	Methylprednisolone, IVIG, and anakinra	No	—	LVSF 34%	Alive
14	Bhardwaj et al.	2022 [21]	22	M	ND	0	ND	ND	ND	25	ND	ND	PEA	—	—	ND	Remdesivir	Steroid	VA-ECMO	5	Fully	Alive
15	Bhardwaj et al.	2022 [21]	53	F	ND	0	ND	ND	ND	5	ND	ND	None	—	—	ND	Remdesivir	Steroid	VA-ECMO	9	LVEF 45%	Alive
16	Bhardwaj et al.	2022 [21]	28	F	ND	0	ND	ND	ND	36	ND	ND	PEA	—	—	ND	Remdesivir	Steroid, IVIG, and tocilizumab	VA-ECMO	5	Fully	Dead
17	Bhardwaj et al.	2022 [21]	27	F	ND	0	ND	ND	ND	22	ND	ND	None	—	—	ND	Remdesivir	Steroid and IVIG	VA-ECMO	10	Fully	Alive
18	Bhardwaj et al.	2022 [21]	46	M	ND	0	ND	ND	ND	8	ND	ND	None	—	—	ND	Remdesivir	Steroid and IVIG	VA-ECMO	6	LVEF 30%	Dead
19	Bhardwaj et al.	2022 [21]	68	M	ND	0	ND	ND	ND	20	ND	ND	None	—	—	ND	No	Steroid	VA-ECMO	2	ND	Alive
20	Bhardwaj et al.	2022 [21]	26	F	ND	1	ND	ND	ND	10	ND	ND	PEA	—	—	ND	No	Steroid	VA-ECMO	9	Fully	Alive
21	Bhardwaj et al.	2022 [21]	66	M	ND	0	ND	ND	ND	10	ND	ND	VT, VF	—	—	ND	No	Steroid	VA-ECMO	8	Fully	Alive
22	Bhardwaj et al.	2022 [21]	24	M	ND	0	ND	ND	ND	15	ND	ND	VF	—	—	ND	No	Steroid	VA-ECMO	7	Fully	Alive
23	Mejia et al.	2022 [22]	17	F	ND	ND	ND	—	No	ND	ND	Yes	None	—	—	ND	No	No	VA-ECMO	10	ND	Alive
24	Rajpal et al.	2022 [23]	60	F	Asthma	ND	Fatigue, shortness of breath, and palpitations	9	No	<15	Yes	Yes	VT	Diffuse hyperintensity on T2 mapping	Scattered perivascular and interstitial inflammatory cells consisting of CD3-positive T-lymphocytes, CD20 positive B-lymphocytes, and histiocytes, along with interstitial and myocyte injury	Yes	No	Methylprednisolone	VA-ECMO	10	Fully	Alive
25	Buitrago et al.	2022 [24]	12	F	None	ND	Headache, neck pain, nausea, diarrhea, and lethargy	2	No	Reduced	No	No	PVC/VT	—	Severe myocarditis without signs of viral infection with severe and diffuse accumulation of CD3 positive T cells	Yes	No	Methylprednisone	VA-ECMO	7	Stable	Alive
26	Thomson et al.	2022 [25]	39	F	Ovarian disease	0	Diarrhoea, vomiting, and abdominal pain	3	No	Near ventricular standstill	Yes	Yes	PEA	—	Mild interstitial infiltrate consisting mostly of CD68+ macrophages along with a lesser number of CD3+ T cells	Yes	No	Methylprednisolone, IVIG, and tocilizumab	VA-ECMO	9	—	Dead
27	Rodriguez Guerra et al.	2022 [26]	56	M	HT and DM	ND	Syncope	<7	No	ND	ND	ND	Long QT	—	—	Yes	No	No	No	1	—	Dead
28	Verma et al.	2022 [27]	48	F	ND	ND	Shortness of breath and chest pressure	5	No	15	Yes	Yes	None	—	Cardiomyocyte damage with prominent macrophage infiltrates. The presence of SARS-CoV-2 in cardiomyocyte is confirmed by RNA scope detecting SARS-CoV-2 spike S antisense strain	Yes	Remdesivir	Methylprednisolone and tocilizumab	VA-ECMO Impella CP	13	Fully	Alive
29	Edwards et al.	2022 [28]	10 months	M	Trisomy 18p, monosomy of 8p, and a small conoventricular ventricular septal defect	ND	Upper respiratory symptoms and fever	4	No	Severely diminished	Yes	Yes	None	T2 weighted imaging demonstrated significantly increased myocardial to skeletal muscle signal intensity	—	Yes	Remdesivir	Dexamethasone and IVIG	VA-ECMO	8	Fully	Alive
30	Aldeghaither et al.	2022 [29]	39	F	None	ND	Fever, dyspnea, chest pain, and diarrhea	28	No	10–15	Yes	No	None	—	Eosinophilic infiltrate of the myocardium	Yes	No	Methylprednisolone	VA-ECMO Impella CP RVAD	9	LVEF of 30–35%	Alive
31	Aldeghaither et al.	2022 [29]	25	M	None	ND	Dyspnea, fever, and hypotension	35	No	15–20	ND	ND	None	—	Mixed inflammatory cells with some eosinophils	Yes	No	Corticosteroid, IVIG, and anakinra	Impella CP	5	LVEF of 35–40%	Alive
32	Aldeghaither et al.	2022 [29]	21	M	ND	ND	Dyspnea and fever	28	No	5–10	ND	ND	None	—	Lymphocytic infiltrate	Yes	No	Methylprednisolone, IVIG, and anakinra	VA-ECMO Impella CP	3	LVEF of 45–50%	Alive
33	Valiton et al.	2022 [30]	52	F	Raynaud syndrome	ND	Shortness of breath, chest pain, and dizziness	3	No	25	Yes	Yes	None	—	Thrombotic microangiopathy of the coronary capillaries with endothelial cell activation (endothelitis) characterized by enlarged nuclei and capillary thrombosis	Yes	No	Dexamethasone	Impella CP	6	Fully	Alive
34	Ismayl et al.	2022 [31]	53	M	None	ND	Fever and upper respiratory symptoms	35	No	25	ND	ND	ND	—	Diffuse interstitial and perivascular neutrophilic and lymphocytic infiltration with rare eosinophils and rare myocyte necrosis	Yes	No	Steroids	VA-ECMO Impella CP	5	Fully	Alive
35	Yalcinkaya et al.	2022 [32]	29	M	ND	0	Chest pain	ND	ND	Reduced ejection fraction	ND	ND	ND	Left ventricular apical thrombus, myocardial edema	Eosinophilic myocarditis	Yes	No	ND	No	—	ND	Alive
36	Nagata et al.	2022 [33]	13	M	None	ND	Fever and malaise, nausea, and watery diarrhea	20	No	20	ND	ND	IRBBB	—	—	Yes	No	Prednisolone and IVIG	No	—	ND	Alive
37	Nishioka and Hoshino	2022 [34]	15	M	None	ND	Fever, fatigue, and abdominal pain	2	No	25	Yes	ND	CAVB, prolonged QT interval, NSVT	—	—	Yes	No	No	VA-ECMO	2	LVEF 45%	Alive
38	Shahrami et al.	2022 [35]	7	M	ND	ND	Dyspnea	10	Yes	25	No	No	AF	—	—	Yes	Hydroxychloroquine	Dexamethasone and IVIG	—	—	LVEF 45%	Dead
39	Menger et al.	2022 [36]	4	F	Obesity	ND	Respiratory distress	7	Yes	ND	ND	ND	ND	—	The posterior wall of the heart showed small-spot fading	Yes	Remdesivir and bamlanivimab	Dexamethasone	VA-ECMO	17	—	Dead
40	Vannella et al.	2021 [37]	26	M	None	ND	Chest pressure, shortness of breath, nausea, vomiting, and chills	7	No	<10	ND	ND	SVT	—	Myocardial necrosis surrounded by cytotoxic T-cells and tissue-repair macrophages	Yes	—	—	VA-ECMO	—	—	Dead
41	Gozar et al.	2021 [38]	3 days	F	No	No	Arrhythmia	2	No	30	Yes	ND	VT	—	—	Yes	—	Dexamethasone and IVIG	No	—	Fully	Alive
42	Shen et al.	2021 [39]	43	M	No	ND	Fever and abdominal pain	49	No	20–25	ND	ND	None	Diffuse myocardial oedema without delayed myocardial enhancement	—	Yes	No	IVIG	IABP	4	Fully	Alive
43	Ishikura et al.	2021 [40]	35	M	No	ND	Fever and general weakness	21	No	7.4	ND	ND	None	—	—	Yes	Yes (details unknown)	Steroid and IVIG	VA-ECMO IABP	7	Fully	Alive
44	Saha et al.	2021 [41]	25 days	F	Sepsis	No	Fever	17	No	40	Yes	No	Cardiac arrest	—	—	Yes	No	Methylprednisolone and IVIG	No	—	LVEF 65%	Alive
45	Yeleti et al.	2021 [42]	25	M	None	ND	Fever, abdominal pain, fatigue, and vomiting	120	No	5–10	Yes	No	VT	Transmural late gadolinium enhancement of basal-mid anterolateral and inferolateral segments	Lymphocytic myocarditis	ND	Remdesivir convalescent plasma	Methylprednisolone	Bilateral Impellas VA-ECMO	3	Normal	Alive
46	Gurin et al.	2021 [43]	26	M	ND	ND	Fevers, chills, headache, nausea, vomiting, and diarrhea	7	Yes	20	Yes	No	None	—	Interstitial edema and inflammatory infiltrate consisting predominantly of interstitial macrophages with scant T-lymphocytes	Yes	—	Solumedrol and IVIG	No	—	LVEF 75%	Alive
47	Fiore et al.	2021 [44]	45	M	None	ND	Shortness of breath, confusion, and asthenia	5	Yes	25	No	No	None	Severe impairment of biventricular global function associated with higher values of T1 and T2 mapping, in the absence of late gadolinium enhancement	Mild lymphohistiocytic inflammatory infiltrate without myocardial necrosis	Yes	Hydroxychloroquine	Anakinra	IABP	7	LVEF 55%	Alive
48	Bemtgen et al.	2021 [45]	18	M	None	ND	Fever, chills, and tachycardia	14	No	25	Yes	No	None	—	Significant infiltration of immune cells (CD68+ macrophages and CD3+ T cells)	Yes	—	Dexamethasone, IVIG, and anakinra	VA-ECMO Impella	7	Fully	Alive
49	Tseng et al.	2021 [46]	5	M	None	No	Fatigue and vomiting	1	No	ND	ND	ND	VT	—	—	Yes	—	Methylprednisolone and IVIG	VA-ECMO	5	ND	Alive
50	Gaudriot et al.	2021 [47]	38	M	Chronic lymphopenia	ND	Chest pain and vomiting	28	Yes	25	Yes	Yes	IRBBB	T2 sequences showed diffuse hyperintense myocardium. Late gadolinium enhancement images demonstrated massive, heterogeneous, and predominantly subepicardial enhancement of the left ventricular myocardium	Myocardial necrosis, suppurated lesions, and lymphocytic infiltration	Yes	—	Antilymphocyte serum, corticosteroids, and mycophenolate mofetil (after heart transplantation)	VA-ECMO Impella	8	Not recovered	Alive (heart transplantation)
51	Menter et al.	2021 [48]	47	F	Obesity	ND	Unconscious and apneic	7	Yes	30	ND	No	VF	—	Mild diffuse necrotizing myocarditis accompanied by extensive thrombotic microangiopathy of cardiac capillaries	Yes	No	No	No	—	No	Dead
52	Ghafoor et al.	2021 [49]	54	F	HT, obesity, and heart failure	ND	Dyspnea, nausea, and vomiting	7	No	10–15	No	No	PEA	—	—	Yes	No	No	VA-ECMO	ND	—	Dead
53	Okor et al.	2021 [50]	72	F	HT and chronic obstructive pulmonary disease	ND	Shortness of breath	7	ND	20	Yes	No	None	—	—	Yes	No	Methylprednisolone	No	—	LVEF 50%	Dead
54	Tomlinson et al.	2021 [51]	13	M	None	ND	Fever, listlessness, abdominal pain, vomiting, diarrhoea, headache, and rash	—	No	53	ND	ND	Ectopic wandering atrial pacemaker	—	—	Yes	No	No	No	—	—	Alive
55	Sampaio et al.	2021 [52]	45	M	None	ND	Dyspnea, fever, myalgia, and postural hypotension	7	Yes	Normal	Yes	No	Asystole	—	—	Yes	Convalescent plasma	Methylprednisolone, IVIG, and tocilizumab	VA-ECMO	9	Fully	Alive
56	Apostolidou et al.	2021 [53]	7	F	Late preterm birth, central hypothyroidism, failure to thrive, and recurrent respiratory tract infections	ND	Headache, loss of appetite, abdominal pain, and vomiting	3	Yes	Fractional shortening 10%	Yes	No	None	—	Acute lymphocytic myocarditis	Yes	Remdesivir convalescent plasma, and interferon-*γ*	Methylprednisolone, anakinra and extracorporeal hemadsorption	VA-ECMO Impella	21	—	Dead
57	Kallel et al.	2021 [54]	26	M	None	ND	Diarrhea, vomiting, fever, fatigue, and weakness	8	Yes	30	Yes	No	None	Normal (7 weeks after the treatment)	—	Yes	No	No	No	—	LVEF 55%	Alive
58	Bulbul et al.	2021 [55]	49	F	None	ND	Cough and shortness of breath	7	Yes	25	ND	ND	None	—	—	Yes	Hydroxychloroquine, oseltamivir, lopinavir, and ritonavir	Methylprednisolone, IVIG, and tocilizumab	VA-ECMO	7	LVEF 50%	Alive
59	Gauchotte et al.	2021 [56]	69	M	HT, DM, and ischemic heart disease	ND	Fever, asthenia, and abdominal pain	7	No	20	Yes	ND	ND	—	Abundant myocardium edema and interstitial inflammation, showing a predominance of mononucleated leucocytes, associated with cardiomyocytes dystrophies	Yes	No	No	VA-ECMO	—	—	Dead
60	Gulersen et al.	2021 [57]	31	F	Pregnant	ND	Cough, myalgias, and diarrhea	7	No	ND	Yes	ND	None	Normal cardiac function (not mentioned about myocarditis)	—	Yes	No	Dexamethasone and IVIG	No	—	Normal	Alive
61	Rasras et al.	2021 [58]	47	F	None	ND	Dyspnea and leg pain	21	Yes	10	ND	ND	ND	—	—	Yes	No	Methylprednisolone	No	—	LVEF 30%	Alive
62	Purdy et al.	2021 [59]	53	M	None	ND	Cough, fever, and shortness of breath	35	No	25	No	No	None	—	—	Yes	Hydroxychloroquine	Methylprednisolone	No	—	LVEF 60%	Alive
63	Purdy et al.	2021 [59]	30	F	Obesity	ND	Fatigue and shortness of breath	9	Yes	45	Yes	ND	None	—	—	Yes	Hydroxychloroquine	Methylprednisolone	No	—	LVEF 55%	Alive
64	Sivalokanathan et al.	2021 [60]	37	M	None	ND	Fever, diarrhea, and dizziness	30	Yes	21	Yes	ND	None	Left ventricular wall thickening, inhomogeneity of T1/T2 mapping values, and patchy non-infarct pattern late gadolinium enhancement in the inferolateral and apical septal walls	—	Yes	No	Hydrocortisone and IVIG	No	—	LVEF 70%	Alive
65	Ruiz et al.	2021 [61]	35	F	Systemic sclerosis	ND	Generalized malaise, fever, and cough	5	Yes	<10	No	No	PEA	Myocarditis (no detail)	—	Yes	Remdesivir	Methylprednisolone and IVIG	Bilateral impellas	14	LVEF 60%	Alive
66	Papageorgiou et al.	2021 [62]	43	M	Mixed connective tissue disease	ND	Fever, cough, and chest pain	4	No	10–15	Yes	No	None	—	No evidence of myocarditis	Yes	No	Hydrocortisone	VA-ECMO Impella CP	7	Normal	Alive
67	Ciuca et al.	2021 [63]	6	M	None	ND	Fever	5	Yes	48	Yes	No	None	Myocardial interstitial edema in T1/T2 mapping	—	Yes	Hydroxychloroquine	Dexamethasone and IVIG	No	—	Fully	Alive
68	Garau et al.	2021 [64]	18	F	None	ND	Nausea and vomiting	1	No	10	Yes	Yes	None	Late gadolinium enhancement in basal to midinferior and inferoseptal segments	A low density of inflammatory cells without myocyte degeneration or necrosis	Yes	Hydroxychloroquine	Methylprednisolone and IVIG	VA-ECMO IABP	17	LVEF 48%	Alive
69	Hékimian et al.	2021 [65]	40	M	Obesity and DM	ND	Dyspnea and asthenia	2	Yes	45	ND	ND	None	—	—	Yes	ND	No	VA-ECMO VV-ECMO	8	LVEF 60%	Alive
70	Hékimian et al.	2021 [65]	19	F	None	ND	Fever, dyspnea, and cough	9	Yes	30	ND	ND	None	—	—	Yes	ND	No	VV-ECMO	15	LVEF 50%	Alive
71	Hékimian et al.	2021 [65]	22	M	Obesity, DM, and asthma	ND	Fever, dyspnea, cough, and asthenia	1	Yes	30	ND	ND	None	—	—	No	ND	No	VV-ECMO	5	LVEF 60%	Alive
72	Hékimian et al.	2021 [65]	19	M	None	ND	Fever, headache, diarrhea, dyspnea, and asthenia	4	No	15	ND	ND	None	—	—	Yes	ND	No	No	—	LVEF 60%	Alive
73	Hékimian et al.	2021 [65]	16	M	None	ND	Fever, anosmia, abdominal pain, rash, conjunctivitis, strawberry tongue, chest pain, asthenia, and adenopathy	7	Yes	20	ND	ND	None	—	—	Yes	ND	IVIG	No	—	LVEF 45%	Alive
74	Hékimian et al.	2021 [65]	17	M	Aortic regurgitation	ND	Fever, headache, abdominal pain, diarrhea, dyspnea, asthenia, and conjunctivitis	4	No	20	ND	ND	None	—	—	Yes	ND	Corticosteroid and IVIG	No	—	LVEF 50%	Alive
75	Hékimian et al.	2021 [65]	17	F	None	ND	Chest pain and dyspnea	1	No	20	ND	ND	VT, cardiac arrest	—	—	Yes	ND	Corticosteroid and IVIG	VA-ECMO	—	No	Dead
76	Milla-Godoy et al.	2021 [66]	45	F	Obesity	ND	Diarrhea, nausea, and vomiting	4	Yes	10	No	ND	Asystole	—	—	Yes	—	Methylprednisolone and IVIG	No	—	No	Dead
77	Hu et al.	2021 [67]	37	M	ND	ND	Chest pain, dyspnea, and diarrhea	3	Yes	27	Yes	ND	None	—	—	Yes	No	Methylprednisoloneand IVIG	No	—	LVEF 66%	Alive
78	Marcinkiewicz et al.	2021 [68]	20	M	None	ND	Fever and dyspnea	42	ND	15	No	Yes	None	Myocardial signal was globally increased on T2-weighted imaging. Delayed late gadolinium imaging showed diffuse fibrosis in the anteroseptal and inferior walls	—	ND	No	No	VA-ECMO IABP	6	LVEF 69%	Alive
79	Gay et al.	2020 [69]	56	M	Obesity, HL	ND	Dyspnea and lethargy	1	Yes	<5	Yes	Yes	ND	—	—	ND	—	Methylprednisolone and tocilizumab	VA-ECMO Impella 2.5/5.0 ProtekDuo	12	LVEF 65%	Alive
80	Jacobs et al.	2020 [70]	48	M	HT	ND	Fever, diarrhea, cough, dysosmia, and dyspnea	7	Yes	ND	No	Yes	ND	—	Hypertrophic cardiac tissue with patchy muscular, sometimes perivascular, and slightly diffuse interstitial mononuclear inflammatory infiltrates, dominated by lymphocytes	Yes	No	—	VA-ECMO	—	No	Dead
81	Lozano Gomez et al.	2020 [71]	53	M	None	ND	Fever and dyspnea	10	No	10	ND	ND	AF	—	—	Yes	No	No	No	—	No	Dead
82	Tiwary et al.	2020 [72]	30	M	HT, DM, chronic kidney disease, glaucoma, and obesity	ND	Abdominal flank pain and shortness of breath	ND	Yes	ND	Yes	ND	LBBB	—	—	Yes	Remdesivir and convalescent plasma	Dexamethasone	No	—	ND	Alive
83	Othenin-Girard et al.	2020 [73]	22	M	None	ND	Asthenia, chills, diffuse myalgia, abdominal pain, and diarrhea	5	No	ND	Yes	ND	CAVB	—	A severe myocardial inflammation with several foci of myocyte necrosis	Yes	—	Methylprednisolone, IVIG, tocilizumab, and cyclophosphamide	VA-ECMO	5	Recovered but not in detail	Alive
84	Albert et al.	2020 [74]	49	M	None	ND	Fevers, myalgias, and dyspnea	14	No	20	ND	Yes	None	—	Mild infiltration of mononuclear cells in the endocardium and myocardium with >14 inflammatory cells per mm2 indicating myocarditis	Yes	No	Methylprednisolone and IVIG	VA-ECMO Impella CP	4	Normal	Alive
85	Salamanca et al.	2020 [75]	44	M	None	ND	Dyspnea and syncope	7	Yes	15	Yes	No	CAVB	Diffuse edema with slightly less involvement of the inferolateral wall on T2 weighted image. T1 mapping with diffuse increase of native T1	Isolated interstitial infiltrate with lymphocytes CD3+	Yes	No	No	VA-ECMO IABP	6	LVEF 75%	Alive
86	Khatri and Wallach	2020 [76]	50	M	HT and ischemic stroke	ND	Fevers, chills, generalized malaise, nonproductive cough, and dyspnea	4	Yes	ND	Yes	ND	None	—	—	Yes	Hydroxychloroquine and methylene blue	Methylprednisolone and IVIG	No	—	No	Dead
87	Bernal-Torres et al.	2020 [77]	38	F	None	ND	Palpitation and general malaise	3	Yes	30	Yes	ND	None	Inflammatory manifestations	—	Yes	Hydroxychloroquine, lopinavir, and ritonavir	Methylprednisolone and IVIG	No	—	LVEF 60%	Alive
88	Chitturi et al.	2020 [78]	65	F	HT, DM, HL, obesity, transient ischaemic attack, and breast cancer	ND	Fever, cough, and shortness of breath	14	Yes	25	Yes	No	None	—	—	Yes	No	Hydrocortisone and tocilizumab	No	—	LVEF 64%	Alive
89	Zeng et al.	2020 [79]	63	M	Allergic cough	ND	Fever, shortness of breath, and chest tightness	ND	Yes	32	No	ND	ND	—	—	Yes	Lopinavir and ritonavir	Methylprednisolone, IVIG, and interferon *α*-1b	VA-ECMO	—	LVEF 68%	Dead
90	Singhavi et al.	2020 [80]	20	M	None	ND	Fever	1	No	30	No	Yes	ND	—	—	Yes	ND	Methylprednisolone	No	—	ND	Alive
91	Naneishvili et al.	2020 [81]	44	F	None	ND	Fever, lethargy, muscle aches, and syncope	3	Yes	37	Yes	Yes	AF	—	—	Yes	No	Methylprednisolone	No	—	Normal	Alive
92	Chao et al.	2020 [82]	49	M	None	ND	Fever and cough	ND	Yes	40	No	No	RBBB	—	—	Yes	Hydroxychloroquine	Tocilizumab	VV-ECMO	12	LVEF 55%	Alive
93	Yan et al.	2020 [83]	44	F	Obesity	ND	Fever, cough, and dyspnea	7	Yes	40	No	No	None	—	Mild myxoid edema, mild myocyte hypertrophy, and focal nuclear pyknosis. Rare foci with few scattered CD45+ lymphocytes	Yes	Hydroxychloroquine	Tocilizumab	No	—	ND	Dead
94	Kesici et al.	2020 [84]	2	M	None	ND	Nausea, vomiting, and poor oral intake	ND	Yes	ND	Yes	No	None	—	—	Yes	ND	ND	VA-ECMO	—	—	Dead
95	Garot et al.	2020 [85]	18	M	None	ND	Cough, fever, fatigue, and myalgias	ND	Yes	30	Yes	Yes	None	Strated nodular subepicardial enhancement of the LV basal posterolateral wall on late gadolinium enhancement images	—	Yes	Hydroxychloroquine	No	No	—	LVEF 54%	Alive
96	Coyle et al.	2020 [86]	57	M	HT	ND	Shortness of breath, fevers, cough, myalgias, decreased appetite, nausea, and diarrhea	7	Yes	35–40	No	No	None	Diffuse biventricular and biatrial edema with a small area of late gadolinium enhancement	—	Yes	Hydroxychloroquine and AT-001 (caficrestat)	Methylprednisolone and tocilizumab	No	—	LVEF 82%	Alive
97	Richard et al.	2020 [87]	28	F	DM, asthma, depression, and intravenous drug use	ND	Lethargy	ND	Yes	26–30	Yes	Yes	RBBB	Myocardial necrosis, fibrosis, and hyperemia, indicating myocarditis	—	Yes	No	Methylprednisolone	Impella	4	LVEF >55%	Alive
98	Pascariello et al.	2020 [88]	19	M	Autistic spectrum disorder	ND	Fever, cough, diarrhea, and vomitting	3	Yes	15–20	ND	ND	None	—	—	Yes	Hydroxychloroquine, remdesivir, and oseltamivir	Dexamethasone	No	—	LVEF 50%	Alive
99	Shah et al.	2020 [89]	19	M	None	ND	Fever, generalized weakness, cough, and shortness of breath	7	Yes	24	No	No	None	—	—	Yes	Hydroxychloroquine	Methylprednisolone, IVIG, and tocilizumab	No	—	LVEF 62%	Alive
100	Veronese et al.	2020 [90]	51	F	Thalassemia minor	ND	Fever, dyspnea, and palpitations	10	No	30	No	Yes	VT, RBBB	Short tau inversion recovery sequences revealed diffuse increased signal intensity suggestive of diffuse edema. Transmural late gadolinium enhancement involved LV basal-lateral and basal-inferior walls	Diffuse lymphocytic inflammatory infiltrates	Yes	No	Methylprednisolone	VA-ECMO IABP	6	Fully	Alive
101	Hussain et al.	2020 [91]	51	M	HT	ND	Fever, cough, fatigue, and dyspnea	ND	Yes	20	No	No	None	—	—	Yes	Hydroxychloroquine	Methylprednisolone	No	—	Not recovered	Alive (ongoing treatment)
102	Gill et al.	2020 [92]	65	F	HT, DM, and breast cancer	ND	Shortness of breath and chest pain	ND	Yes	25	No	No	None	—	—	Yes	—	—	IABP	—	No	Dead
103	Gill et al.	2020 [92]	34	F	None	ND	Shortness of breath, chest pain, and weakness	ND	No	20	Yes	No	None	—	—	Yes	—	Methylprednisolone	VA-ECMO	4	LVEF 60%	Alive
104	Fried et al.	2020 [93]	64	F	HT, HL	ND	Chest pressure	2	No	30	Yes	Yes	None	—	—	Yes	Hydroxychloroquine	No	IABP	7	LVEF 50%	Alive
105	Craver et al.	2020 [94]	17	M	None	ND	Headache, dizziness, nausea, and vomiting	2	No	ND	ND	ND	Asystole	—	Diffuse inflammatory infiltrates composed of lymphocytes, macrophages, with prominent eosinophils	ND	—	—	—	—	—	Dead
106	Irabien-Ortiz et al.	2020 [95]	59	F	HT, cervical degenerative arthropathy, chronic lumbar radiculopathy, lymph node tuberculosis, and migraine	ND	Fever and chest pain	5	No	Preserved	Yes	Yes	Asystole	—	—	Yes	IFN B, lopinavir, and ritonavir	Methylprednisolone and IVIG	VA-ECMO IABP	ND	Fully	Alive (ongoing treatment)
107	Tavazzi et al.	2020 [96]	69	M	ND	ND	Dyspnoea, persistent cough, and weakness	4	Yes	25	No	No	ND	—	Low grade interstitial and endocardial inflammation	Yes	—	—	VA-ECMO IABP	5	Not recovered	Dead
108	Gomila-Grange et al.	2020 [97]	39	M	ND	ND	Fever, right flank pain, and diarrhea	6	Yes	20	Yes	No	None	—	—	Yes	Hydroxychloroquine	Tocilizumab	No	—	Normal	Alive

AF, atrial fibrillation; CAVB, complete atrioventricular block; CMR, cardiovascular magnetic resonance; DM, diabetes mellitus; F, female; HL, hyperlipidemia; HT, hypertension; IABP, intra-aortic balloon pumping; IRBBB, incomplete right bundle branch block; IVIG, intravenous immunoglobulin; LGE, late gadolinium enhancement; LV, left ventricle; LVEF, left ventricular ejection fraction; LVSF, left ventricular shortening fraction; LVWT, left ventricular wall thickening; M, male; MCS; mechanical circulatory support; ND, not described; NSVT, nonsustained ventricular tachycardia; PE, pericardial effusion; PEA, pulseless electrical activity; PVC, premature ventricular contraction; VA/VV-ECMO, veno-arterial/veno-venous extracorporeal membrane oxygenation; VF, ventricular fibrillation; and VT, ventricular tachycardia.

**Table 2:** Demographics and clinical data of studied patients. All descriptive parameters are obtained from the original papers.

Demographic variables	

	*N* = 108
*Age (years)*	34.8 ± 18.1 (range 0–72)
≤20	30 (27.8%)
≥60	10 (9.3%)
*Sex*	
Male	67 (62.0%)
Female	41 (38.0%)
*Comorbidities*	
Not described	21
None	48
Hypertension	12
Obesity	11
Diabetes mellitus	8
Asthma (including allergic cough)	4
Heart diseases	4
Gynecologic diseases	3
Hyperlipidemia	3
Connective tissue disorders	3
Blood disorders	2
Mental disorders	2

*Dose of vaccination*	*N* *=* *19*
0	15
1	2
2	1
3	1
≥4	0

Clinical data	

*Initial symptoms*	*N* *=* *98 (excluding 10 patients with relevant information unavailable)*
Fever	51 (52.0%)
Dyspnea or shortness of breath	45 (45.9%)
Diarrhea	20 (20.4%)
Chest pain	20 (20.4%)
Cough	19 (19.4%)
Vomiting	17 (17.3%)
Abdominal pain	13 (13.3%)
Asthenia	9 (9.2%)
Fatigue	9 (9.2%)
Weakness	5 (5.1%)
Lethargy	5 (5.1%)
Loss of appetite	3 (3.1%)

*Concurrent with pneumonia*	*N* *=* *43*
2020	20
2021	20
2022	3

*Left ventricular ejection fraction (LVEF)*	*N* *=* *108*
LVEF ≤ 20%	48 (52.2%)
20 < LVEF ≤ 30%	31 (33.7%)
30 < LVEF ≤ 40%	7 (7.6%)
40 < LVEF ≤ 50%	3 (3.3%)
50% < LVEF including preserved or normal	3 (3.3%)
Unclassified	16

*Pericardial effusion*	*N* *=* *108*
Yes	45 (65.2%)
No	24 (34.8%)
Not described	39
*Left ventricular wall thickening*	*N* *=* *108*
Yes	24 (40.7%)
No	35 (59.3%)
Not described	49

*Arrhythmia*	*N* *=* *40*
VT	11
Asystole/cardiac arrest	6
PEA	6
VF	5
RBBB	5
AF	4
CAVB	4
Long QT	2
Ectopic wandering atrial pacemaker	1

*Diagnostic modality*	*N* *=* *49*
Only CMR	14
Only biopsy	23
Both CMR and biopsy	12

*Mechanical circulatory support*	*N* *=* *67*
ECMO	56 (83.6%)
Impella	19 (28.4%)
IABP	12 (12.9%)
RVAD	2 (3.0%)
Combination	20 (29.9%)

*Outcome*	*N* *=* *107 (excluding 1 patient with relevant information unavailable)*
Alive	83 (77.6%)
Dead	24 (22.4%)

AF, atrial fibrillation; CAVB, complete atrioventricular block; CMR, cardiovascular magnetic resonance; ECMO, extracorporeal membrane oxygenation; IABP, intra-aortic balloon pumping; LVEF, left ventricular ejection fraction; PEA, pulseless electrical activity; RVAD, right ventricular assist device; RBBB, right bundle branch block; VF, ventricular fibrillation; and VT, ventricular tachycardia.

## Abbreviations

CMR: Cardiac magnetic resonance
COVID-19: Coronavirus disease 2019
EAT: Epicardial adipose tissue
ECMO: Extracorporeal membrane oxygenation
EMB: Endomyocardial biopsy
IABP: Intra-aortic balloon pumping
IL-6: Interleukin 6
IVIG: Intravenous immunoglobulin
LVEF: Left ventricular ejection fraction
MCS: Mechanical circulatory support
PRISMA: Preferred Reporting Items for Systematic Reviews and Meta-Analysis
RVAD: Right ventricular assist device
SARS-CoV-2: Acute respiratory syndrome coronavirus 2.
